# BAP effect in vitro germination of soybean cultivars UFUS Xavante an UFUS Carajás

**DOI:** 10.1186/1753-6561-8-S4-P91

**Published:** 2014-10-01

**Authors:** Ana Paula Rodrigues Gomes, Ana Paula Oliveira Nogueira, Giovanna Dias De Sá, Bárbara Rodrigues, Ana Maria Bonetti

**Affiliations:** 1 Universidade Federal de Uberlândia, Uberlândia, Brazil

## Background

Plant tissue culture is a biotechnology particularly relevant to plant genetic transformation. The ability of in vitro regeneration is one of the requirements for the production of usable plants in breeding programs [1]. As each genotype has a specific regeneration potential, several protocols have been developed with the aim of developing methodologies that can speed up the germination process [2]. *In vitro*, culture media for plants, not only provide macro and micronutrients, but also carbohydrates, usually sucrose as a carbon source. Better results are obtained by adding organic compounds such as vitamins, amino acids and growth regulators [3].

## Methods

This study evaluated the germination of seeds of two soybean cultivars on MS medium with different concentrations of 0, 1, 3 and 5 µg / L BAP. The cultivars used were UFUS Xavante and UFUS-Carajás from the Breeding Program Soybean by Federal University of Uberlândia. Seeds were placed in a solution of alcohol/water 70% (v/v) for 1 min and then immersed in 0.5% sodium hypochlorite (w/v) for 30 min. After surface sterilization, the seeds will be washed with distilled water and autoclaved. After desinfestation (quoting the type of disinfection) seeds of cultivars were soaked in distilled water autoclaved for a period of 1 and 7 days. Subsequently, the seeds were inoculated in test tubes with the medium remains under dark for 7 days. The evaluations were done every 7 days for 28 days [4].

## Results and conclusions

We evaluated the occurrence of rupture of the radicle and seedling formation. After 7 days it was observed that the cultivar UFUS-Xavante that were soaked in water for one day and inoculated in medium with 3 µg / L BAP showed 100% of germination. On the other hand, the soaking for 7 days led to a decrease in germination percentage (80%) independent of the hormone concentration. At 14 days, there was the emergence of the cotyledons for both cultivars in medium with 3 µg / L BAP and with 21 days, there was the formation of seedlings in this regulator concentration. It was observed that increasing the regulator concentration associated with prolonged soaking conditions were not favorable for both varieties. New regulators combinations are necessary to assess the effect of the growth regulator and the time soybean's formation seedlings in vitro.
